# Progressive multifocal leukoencephalopathy or immune reconstitution inflammatory syndrome after fingolimod cessation? A case report

**DOI:** 10.1186/s12883-022-02839-3

**Published:** 2022-08-19

**Authors:** D. Mickeviciene, A. Baltusiene, B. Afanasjeva, D. Afanasjevas, R. Gleizniene, D. Rastenyte, JR. Berger

**Affiliations:** 1 Department of Neurology, Hospital of Lithuanian University of Health Sciences Kauno Klinikos, Kaunas, Lithuania; 2 Department of Neurology, Lithuanian University of Health Sciences Clinical, Kaunas, Lithuania; 3 Faculty of Medicine, Lithuanian University of Health Sciences, Kaunas, Lithuania; 4 Department of Radiology, Lithuanian University of Health Sciences Clinical, Kaunas, Lithuania; 5 Perelman School of Medicine, University of Pennsylvania, Philadelphia, PA USA

**Keywords:** Multiple sclerosis, Fingolimod, Immune reconstitution inflammatory syndrome, Progressive multifocal leukoencephalopathy, Case report

## Abstract

**Background:**

Fingolimod is associated with an increased risk of developing progressive multifocal leukoencephalopathy (PML); however, its discontinuation may cause severe immune reconstitution inflammatory syndrome (IRIS). As both of these conditions (especially fingolimod induced PML) are rarely described in medical case reports distinguishing between PML-IRIS and MS-IRIS may be diagnostically challenging.

**Case presentation:**

We report a patient with severe clinical decline (Expanded Disability Status Scale (EDSS) increasing from 3.5 to 7.5) and multiple, large, contrast-enhancing lesions on brain magnetic resonance imaging (MRI) a few months after fingolimod withdrawal. The diagnostic possibilities included IRIS due to fingolimod withdrawal versus PML-IRIS. The JC virus (JCV) antibody index was positive (2.56); however, cerebrospinal fluid (CSF) JCV real-time polymerase chain reaction (JCV-PCR) was negative and brain biopsy was not performed. After a long course of aggressive treatment (several pulsed methylprednisolone infusions, plasmapheresis, intravenous dexamethasone, oral mirtazapine) the patient gradually recovered (EDSS 2.5) and MRI lesions decreased.

**Conclusions:**

This case report demonstrates the importance of monitoring patients carefully after the discontinuation of fingolimod for PML-IRIS and rebound MS with IRIS as these conditions may manifest similarly.

## Background

Many disease-modifying therapies (DMTs) used in the treatment of multiple sclerosis (MS) are associated with immunosuppression and an increased risk of developing progressive multifocal leukoencephalopathy (PML) [1]. The risk of PML is the greatest with natalizumab [1]; however, it has also been observed with fingolimod and other DMTs. The estimated risk of PML with fingolimod is 0.069 per 1,000 patients and the estimated incidence rate is 3.12 per 100,000 patients-years [2]. Discerning PML from MS rebound occurring on the heels of fingolimod cessation, a phenomenon attributed to severe immune reconstitution inflammatory syndrome (IRIS) [3] can be diagnostically challenging. We report a patient developing clinical and magnetic resonance imaging (MRI) findings at the time of fingolimod discontinuation that resulted in this diagnostic dilemma.

## Case presentation

A 26-year-old woman was suspected of having MS in 2011. The following year a diagnosis of relapsing–remitting MS (RRMS) was confirmed and recombinant human interferon beta-1a initiated. Following two relapses accompanied by brain MRI, interferon β1a was discontinued and fingolimod initiated. At that time, her JC virus (JCV) antibody index was positive at 2.56. During fingolimod treatment, she exhibited lymphocytopenia (lymphocyte count 0.3–0.8 × 10^9^ per liter) and experienced a few mild opportunistic infections including (shingles and stomatitis; however fingolimod therapy was not interrupted. She had two relapses while on fingolimod including internuclear ophthalmoplegia in one instance and right hemihypesthesia in the other. Both resolved following treatment with pulsed intravenous methylprednisolone 1000 mg. The disability assessed by Expanded Disability Status Scale (EDSS) during fingolimod treatment was 2—2.5.

**Fig. 1:**
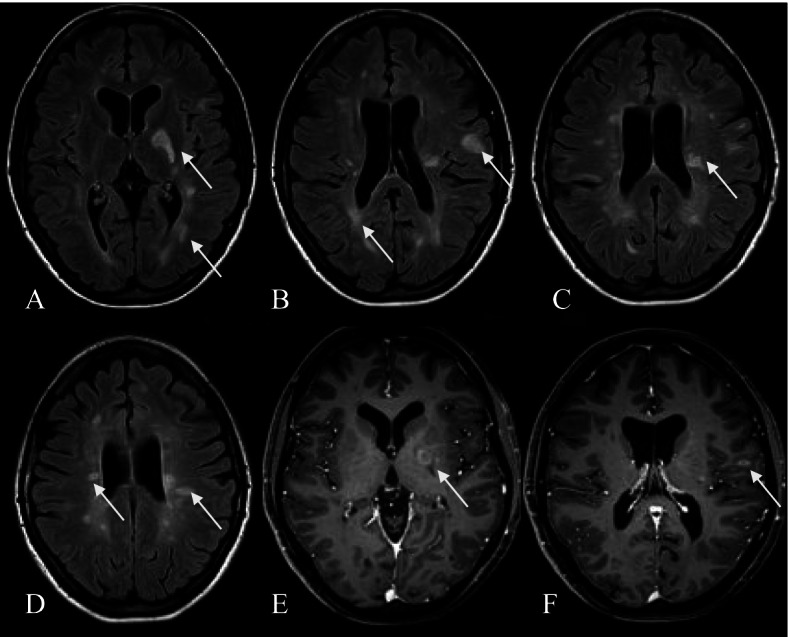
MRI of the patient (2020.09.20). **A-D** axial T2W FLAIR presents new hyperintense lesions (arrows). **E**–**F** T1W with contrast shows new gadolinium-enhancing lesions (arrows)

On the August 24, 2020, the patient discontinued fingolimod to attempt conception. The patient made this decision on her own despite the recommendations of her treating physicians. On the September 20, 2020, her brain MRI (Fig. 1) showed signs of disease activity and progression (more than five new hyperintense lesions on T2-weighted images, a few new gadolinium-enhancing lesions).

The patient was informed about the risks associated with disease and disability progression; nevertheless, she refused to continue DMT and delay pregnancy. Pulse therapy with methylprednisolone was given for three days; and her neurologic condition stabilized. On the October 3, 2020, the patient presented to the emergency department with right facial paralysis and received three days course of pulsed methylprednisolone. Partial recovery after treatment was achieved, but on the October 21, 2020, the right facial paralysis worsened and was accompanied by dysarthia and dysphagia. Two days later the patient was hospitalized.

**Fig. 2:**
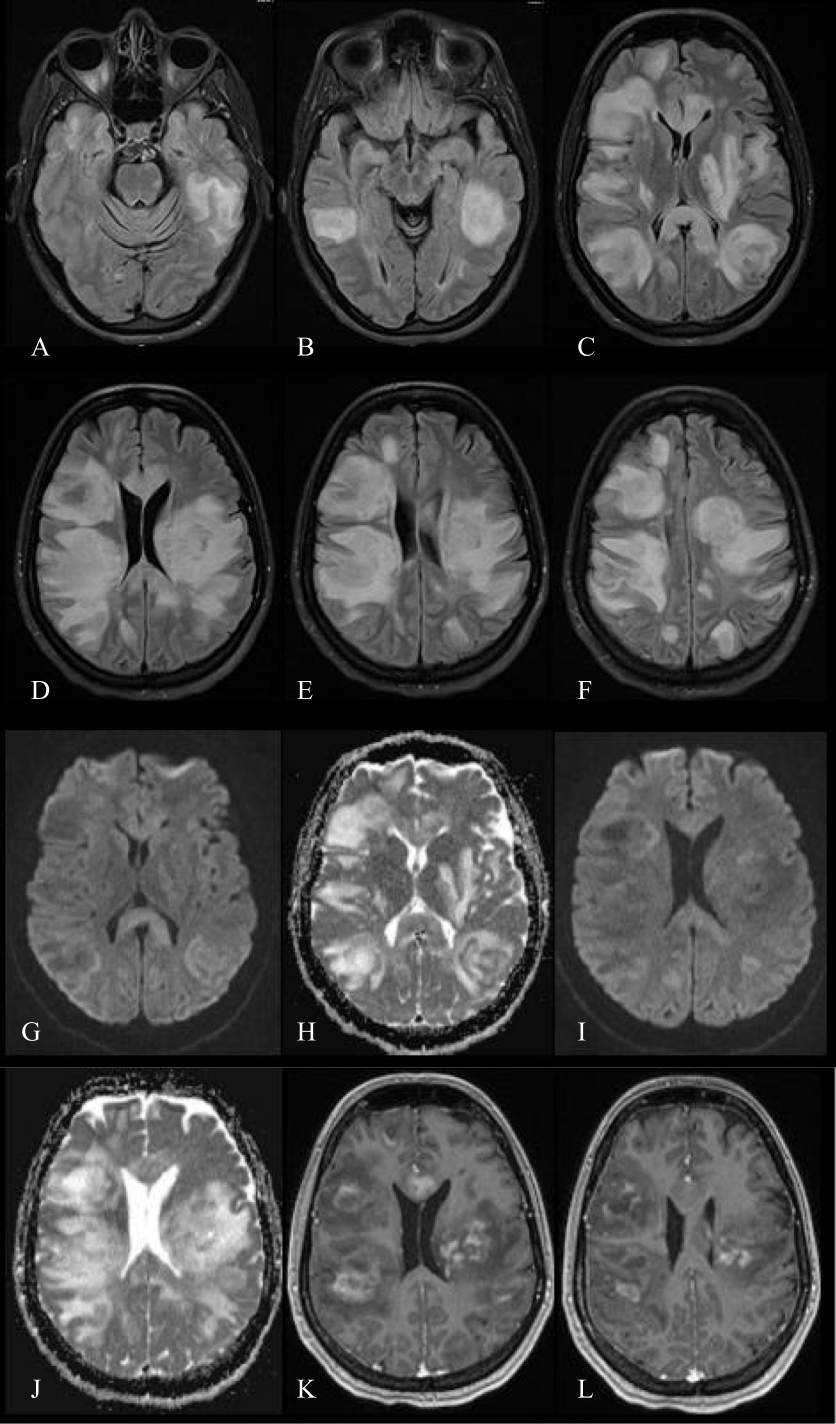
Follow-up MRI of the patient (2020.11.06) with further expansion of the lesions, broad edematous areas and ventricular compression. **A-F** axial T2W FLAIR images; **G**, **I** axial DW; **H**, **J** axial ADC; **K**, **L** axial T1W with contrast

On admission, neurological examination revealed right facial hemiparesis, dysphonia, intermittent dysphagia, absent gag reflex, pathological Babinski reflex on the right and ataxia with right limbs. Laboratory testing showed mild lymphopenia (lymphocyte count 0.7 × 10^9^ per liter). Three days of high dose methylprednisolone was administered intravenously, rehabilitation initiated. No clinical improvement was observed and therapeutic plasmapheresis therapy was initiated. Despite these efforts, the patient continued to worsen with progressive speech impairment and dysphagia (requiring the insertion of a nasogastric tube for nutrition), disorientation and confusion. The working diagnosis was MS-IRIS, but her inexorable progression despite aggressive treatment raised concerns for PML. A brain MRI from October 28, 2020, revealed enlargement of previously detected lesions and new hyperintense T2W FLAIR lesions with uneven gadolinium-enhancement mostly in the periphery; magnetic resonance spectroscopy showed a lactate peak with decreased N-acetyl aspartate, increased choline and creatine peaks. Some lesions were suggestive of PML but overall, the findings were believed to be most consistent with rebound MS. Despite more aggressive plasmaphereses, her disease worsened. Examination revealed global aphasia, an altered mental status, left hemiparesis; she became unresponsive and bedridden with complete incontinence. Repeated brain MRI showed further expansion of her lesions with broad edematous areas and ventricular compression (Fig. 2).

**Fig. 3:**
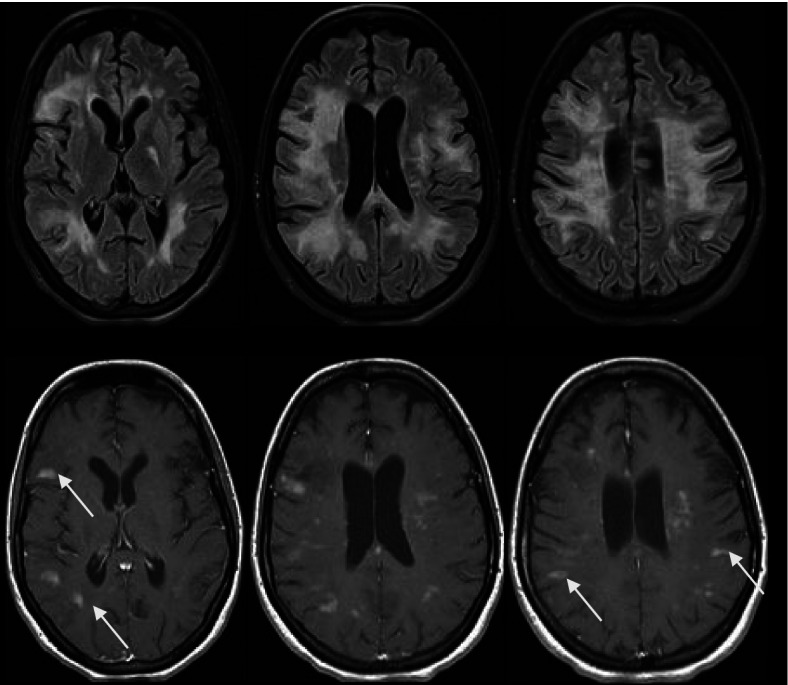
Another follow-up MRI of the patient (2020.11.24). **A-C** axial T2W FLAIR showed decreasing lesions and diminishing mass effect as well as new hyperintense lesions; **D-F** axial T1W with contrast revealed gadolinium enhancement in new lesions (arrows)

The diagnostic possibilities for these radiological findings of broad edematous, gadolinium enhancing lesions included MS-IRIS due to fingolimod withdrawal versus PML-IRIS. Lumbar puncture was performed, but cerebrospinal fluid (CSF) examination showed no signs of infection and JCV real-time polymerase chain reaction (JCV-PCR) was negative. Her JCV antibody index remained nearly the same (2.57). Biopsy, which was not pursued, was the only reliable method to distinguish between these two possibilities as a negative CSF JCV PCR was insufficiently sensitive to entirely exclude PML. Because of concern about possible PML, mirtazapine 15 mg a day was initiated. She was also started on intravenous dexamethasone 32 mg daily. Within several days of this regimen, her condition began to improve and she was able to sit, began walking with assistance, and started to communicate by signs with medical staff, although she remained mute and highly dependent. Lymphopenia worsened (from 0.7 to 0.1 × 10^9^ per liter) which was attributed to the corticosteroid administration. Intravenous immunoglobulin (3 doses; a total of 30 g) was administered because of concern about secondary immunodeficiency and to prevent infectious complications. However, with the lowest levels of lymphocyte counts, the patient suffered another clinical decline and was diagnosed with staphylococcal sepsis (*S. aureus*) and pneumonia requiring antimicrobial therapy. Repeat brain MRI (Fig. 3) performed 18 days after the last revealed decreasing lesions and diminishing mass effect as well as new, gadolinium-enhancing T2W FLAIR hyperintense lesions in cerebral hemispheres and brainstem.

Another course of pulsed methylprednisolone therapy was administered after the antibiotics therapy had been completed and her lymphocyte count normalized. Gradual improvement to full independence eventually occurred with complete resolution of aphasia, dysarthria, dysphagia, hemiparesis and ataxia; however, her cognitive functions remained impaired.

On December 10, 2020, the patient was discharged from the hospital. A brain MRI from January 9, 2021, showed a decrease in the size of the lesions, an improvement in the mass effect and fewer gadolinium-enhancing zones lesions. As of June 2021, she is clinically stable with impaired (but gradually improving) cognitive functions on ocrelizumab.

## Discussion

Fingolimod is a sphingosine-1-phosphate receptor modulator. It is a disease-modifying drug approved for the treatment of relapsing–remitting multiple sclerosis. Fingolimod reduces the activity of multiple sclerosis by reducing circulating lymphocyte counts, preventing lymphocytes from entering the central nervous system (CNS) [4]. The use of this drug increases the risk of developing PML and the abrupt discontinuation of fingolimod may lead to immune reconstitution inflammatory syndrome (IRIS) [4, 5]. The case we present reflects the concern that may arise in distinguishing between MS-IRIS and PML-IRIS.

Lymphocytopenia is a common finding in fingolimod treatment; lymphocyte counts usually return to normal ranges within 4 to 8 weeks after fingolimod discontinuation. Lymphocyte reuptake is one of the main signs of disease’s reactivation, and a sudden rise in lymphocytes may be a portent for worsening MS [6]. During the treatment with fingolimod, the leukocyte count in the blood of our clinical case fluctuated from 0.3 to 0.8 × 10^9^/l; lymphocyte count was 0.36 × 10^9^/l immediately after discontinuation of fingolimod and 0.85 × 10^9^/l 6 weeks later. Severe lymphocytopenia (0.1 × 10^9^/l) was observed during hospitalization but recovered to 1.0 × 10^9^/l after treatment (after administration of intravenous immunoglobulin). However, in our case, the fluctuations in lymphocyte counts could not be solely attributed to fingolimod, as the patient was treated with glucocorticoids and plasmapheresis, both of which may affect lymphocyte counts.

IRIS occurs in 10 per cent of patients with MS who discontinued fingolimod [3, 7]. IRIS manifests as a severe neurological condition with atypical large-scale lesions on MRI. The median time from cessation to requiring symptoms is 2–4 months [8]. Our patient discontinued treatment at the end of August, and on repeated MRI in late September, an increase in lesion burden was observed, but at that time, the patient had no new symptoms. A month later, she acutely deteriorated. The combination of clinical and radiographic worsening shortly after fingolimod deterioration suggested rebound MS, an IRIS phenomenon. Glucocorticoids and plasmaphereses are recommended for the treatment of IRIS; rituximab treatment is sometimes used as well [8]. Our patient was treated with glucocorticoids and plasmapheresis therapy.

PML is a demyelinating CNS disease caused by the reactivation of the JC virus. Seroepidemiological studies indicate that JCV generally acquired in childhood. More than 50% of the adult population worldwide has antibody to JCV [1]. PML most commonly occurs in the presence of immunosuppression (HIV, hematologic disorders, immunosuppressive medications). Of all disease-modifying drugs for multiple sclerosis natalizumab has the highest and most well-known risk of developing PML, but fingolimod has also been associated with PML in 37 reported cases [1, 2, 9]. The clinical signs characteristic of PML are very diverse: speech, consciousness, orientation and coordination disorders, paresis, and others [1, 10]. The nature of the neurological deficits our patient experienced, particularly, cognitive and language disturbances raised concerns about PML as these are rare in MS [11]. However, the rapidity with which they developed were more consistent with rebound MS. The radiographic imaging appearance of the lesions and the pattern of their enhancement did not clearly distinguish between PML-IRIS [12] or MS IRIS (alternately referred to as "rebound MS with severe inflammatory reactions") due to interruption of fingolimod therapy. The lesions did not appear to extend in a tract-dependent fashion as has been described in PML [13], favoring the diagnosis of MS-IRIS. The absence of evidence of JCV infection in CSF by PCR or on in tissue precluded the diagnosis of “definite” PML [12]. Her clinical features, especially her eventual improvement, negative CSF JCV-PCR, and imaging findings, were more supportive of rebound MS with IRIS rather than PML-IRIS as the diagnosis. While plasmaphereses and glucocorticoids are commonly used to treat PML-IRIS, their use and that of mirtazapine remains anecdotal [1, 10] and currently unsupported by randomized controlled trials [14, 15]. Furthermore, PML-IRIS can be the consequence of rapid drug elimination by plasmaphereses. PML-IRIS is one of the reasons why the use of PLEX in natalizumab PML remains controversial. Nonetheless, an empiric trial of glucocorticoids, plasmaphereses, and mirtazapine were initiated in our patient.

## Conclusion

This case report demonstrates the importance of monitoring patients carefully after the discontinuation of fingolimod for PML-IRIS and rebound MS with IRIS as these conditions may manifest similarly. The evaluation of the clinical condition, progression of the disease, cerebral MRI findings and cerebrospinal fluid play the major role in differentiating between IRIS and PML.

## Data Availability

The datasets analyzed during the current study are not publicly available due to privacy reasons but are available from the corresponding author on reasonable request.

## Abbreviations

CNS: Central nervous system
CSF: Cerebrospinal fluid
DMTs: Disease-modifying therapies
EDSS: Expanded Disability Status Scale
IRIS: Immune reconstitution inflammatory syndrome
JCV: JC virus
MS: Multiple sclerosis
MS-IRIS: Multiple sclerosis with immune reconstitution inflammatory syndrome
MRI: Magnetic resonance imaging
PCR: Real-time polymerase chain reaction
PML: Progressive multifocal leukoencephalopathy
PML-IRIS: Inflammatory form of progressive multifocal leukoencephalopathy
RRMS: Relapsing–remitting form of multiple sclerosis

## References

[1] Mills EA, Mao-Draayer Y (2018). Understanding progressive multifocal leukoencephalopathy risk in multiple sclerosis patients treated with immunomodulatory therapies: A bird’s eye view. Front Immunol..

[2] Berger JR, Cree BA, Greenberg B, Hemmer B, Ward BJ, Dong VM (2018). Progressive multifocal leukoencephalopathy after fingolimod treatment. Neurology.

[3] Evangelopoulos ME, Miclea A, Schrewe L, Briner M, Salmen A, Engelhardt B (2018). Frequency and clinical characteristics of Multiple Sclerosis rebounds after withdrawal of Fingolimod. CNS Neurosci Ther.

[4] Gyang TV, Hamel J, Goodman AD, Gross RA, Samkoff L (2016). Fingolimod-associated PML in a patient with prior immunosuppression. Neurology.

[5] Tan TS, Koralnik IJ (2010). Progressive multifocal leukoencephalopathy and other disorders caused by JC virus: clinical features and pathogenesis.

[6] Ghadiri M, Fitz-Gerald L, Rezk A, Li R, Nyirenda M, Haegert D, Giacomini PS, Bar-Or A, Antel J (2017). Reconstitution of the peripheral immune repertoire following withdrawal of fingolimod. Mult Scler..

[7] De Mercanti SF, Gned D, Matta M, Iudicello M, Franchin E, Clerico M (2020). Atypical Multiple Sclerosis Lesions or Progressive Multifocal Leukoencephalopathy Lesions: That Is the Question. J Investig Med High Impact Case Rep.

[8] Barry B, Erwin AA, Stevens J, Tornatore C (2019). Fingolimod Rebound: A Review of the Clinical Experience and Management Considerations. Neurol Ther.

[9] R. Fox, B. Cree, B. Greenberg, B. Hemmer, B.J. Ward, D. Ontaneda, A. Moore, Y. Zhang, R. Sullivan, P. Girase, T. Hach, J.R. Berger. Update on the risk estimates of progressive multifocal leukoencephalopathy related to fingolimod. Oral presentation at the 8thJoint ACTRIMS-ECTRIMS Meeting, MSVirtual2020, September 11‒13, 2020. https://www.medcommshydhosting.com/MSKnowledgecenter/ectrims/presentations/CPO/FC02.02.pdf.

[10] Cortese I, Reich DS, Nath A (2021). Progressive multifocal leukoencephalopathy and the spectrum of JC virus-related disease. Nat Rev Neurol.

[11] Yoshii F, Moriya Y, Ohnuki T, Ryo M, Takahashi W (2017). Neurological safety of fingolimod: An updated review’’. Clin Exp Neuroimmunol.

[12] Wattjes MP, Wijburg MT, Vennegoor A, Witte BI, de Vos M, Richert ND, Uitdehaag BMJ, Barkhof F, Killestein J (2016). MRI characteristics of early PML-IRIS after natalizumab treatment in patients with MS. J Neurol Neurosurg Psychiatry..

[13] Ono D, Shishido-Hara Y, Mizutani S, Mori Y, Ichinose K, Watanabe M, Tanizawa T, Yokota T, Uchihara T, Fujigasaki H (2019). Development of demyelinating lesions in progressive multifocal leukoencephalopathy (PML): Comparison of magnetic resonance images and neuropathology of post-mortem brain. Neuropathology.

[14] Berger JR (2009). Steroids for PML-IRIS: a double-edged sword?. Neurology.

[15] Landi D, De Rossi N, Zagaglia S, Scarpazza C, Prosperini L, Albanese M, Buttari F, Mori F, Marfia GA, Sormani MP, Capra R, Centonze R (2017). No evidence of beneficial effects of plasmapheresis in natalizumab-associated PML. Neurology.

